# Social network interventions in the space of topological relationships between communities

**DOI:** 10.1007/s13278-022-00976-8

**Published:** 2022-10-21

**Authors:** Padraig Corcoran, Philipp Reinecke, Martin Innes

**Affiliations:** 1 School of Computer Science and Informatics, Cardiff University, Cardiff, UK; 2 Crime and Intelligence Innovation Institute, Cardiff University, Cardiff, UK

**Keywords:** Social network, Community, Topological relationship, Intervention

## Abstract

A social network intervention is a process of intentionally altering a social network to achieve an objective. The objective in question may concern accelerating behaviour change or improving organisational performance. In this work we propose a novel model of social network interventions which considers topological properties of relationships existing between communities. Broadly speaking, topological properties of such relationships include properties described by natural language descriptions such as contains, partial overlap and disjoint. The proposed model provides an abstraction which in many cases is useful for solving problems involving social network interventions. We demonstrate this by simulating interventions on a number of hypothetical and real social networks in the domains of health and security.

## Introduction

A *social network* is a graph-based model where vertices model individuals and edges model the existence of relationships between individuals. The type of relationship modelled by a given social network varies and is a function of the application in question. However, three commonly modelled relationships are friendship, social influence and information/resource flow. Social network modelling has successfully been applied in many application domains. For example, De la Haye et al. (2010) and Ranciati et al. (2020) performed social network modelling of obesity-related behaviours and terrorist activities, respectively.

A social network intervention is a process of intentionally altering a social network to help achieve some objective. Here altering a social network includes actions such as adding or deleting an edge in the social network. The objective in question may relate to accelerating behaviour change or improving organisational performance (Valente 2012, 2017). Many studies have demonstrated the usefulness of social network interventions (Hunter et al. 2019). Wang et al. (2011) demonstrated interventions to be useful for promoting condom use. Spencer-Bonilla et al. (2017) demonstrated interventions to be useful for improving social support and glycaemic control in patients with type 2 diabetes. Sciabolazza et al. (2020) demonstrated interventions to be useful for fostering scientific collaboration at a research university. Finally, we are unfortunately too well aware of the interventions, of reducing face-to-face interactions, performed by many governments to reduce COVID-19 transmission (Centola 2020).

Valente (2012) defined a set of models of social network interventions which involve making alterations to the social network by considering vertices, edges and individual communities. A community is defined as a subset of individuals who share a particular characteristic. For example, in the area of security one may be interested in modelling communities corresponding to individuals belonging to terrorist groups and national security agencies. In this work we propose a novel model of social network interventions which considers topological properties of relationships existing between communities. We refer to such relationships as *topological relationships*. Relationships existing between communities can exhibit many different properties. Broadly speaking, *topological properties* of such relationships include properties described by natural language descriptions such as *contains*, *partial overlap* or *disjoint*. The proposed model represents a generalisation of the original models proposed by Valente (2012). Specifically, the proposed model provides a higher-level abstraction which in many cases is more useful or suitable for solving problems involving social network interventions.

**Fig. 1:**
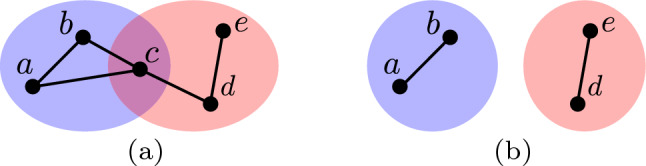
Hypothetical social networks containing blue and red communities are displayed in (**a**) and (**b**). The communities exhibit topological relationships of *partial overlap* and *disjoint*, respectively (colour figure online)

To illustrate this point consider a social network which contains one community corresponding to terrorists and a second community corresponding to individuals with access to and skills in the use of weapons. Furthermore, let us say these communities are modelled by the social network displayed in Fig. 1(a) where individuals belonging to the former community lie in the blue coloured region, while individuals belonging to the latter community lie in the red coloured region. In this social network, the existence of an edge between two vertices indicates the flow of information between the individuals in question. Due to the fact that individual *c* belongs to both communities, the relationship between the communities may be described as *partial overlap*. Such a relationship may be considered a *security threat* because the community with access to and skills in the use of weapons may share this with the terrorist community. Modelling the topological relationship between these communities allows the detection of this threat. Furthermore, the ability to apply an intervention which considers the topological relationship allows the elimination of this threat. For example, if one could apply an intervention to the social network in Fig. 1(a) to remove the individual *c*, the social network would change to that in Fig. 1(b). That is, the relationship between the communities would change from *partial overlap* to *disjoint* and in turn the security threat would be eliminated. The operation of removing individual *c* could be performed by detaining the individual in question.

By defining interventions in terms of topological relationships we abstract away details relating to individual vertices, edges and communities which are unnecessary for solving many problems. For example, when modelling the relationships of *partial overlap* or *disjoint* we can abstract away which vertices belong to which communities or how these vertices are connected by edges. Instead, we can model both relationships as a corresponding binary variable indicating the presence or absence of the relationship in question. The use of this higher-level abstraction can therefore in many cases make the process of reasoning about intervention less complex. Note that, an intervention defined in terms of topological relationships will ultimately be implemented in terms of interventions defined in terms of vertices, edges and individual communities. For example, performing the intervention in the above example which transforms the relationship from *partial overlap* to *disjoint* is ultimately implemented by removing the individual or vertex *c*. The model proposed in this work can take an intervention defined in terms of topological relationships and determine a corresponding implementation.

The remainder of this paper is structured as follows. In Sect. 2 we review related works on social network communities, social network interventions and modelling topological relationships. In Sect. 3 we present the proposed model of social network interventions which considers topological relationships between communities. In Sect. 4 we demonstrate that the proposed model provides a useful abstraction for solving many real-world problems involving social network interventions. This is achieved by simulating interventions on a number of hypothetical and real social networks. Finally, in Sect. 5 we draw conclusions from this work and discuss possible directions for future research.

## Related works

Social networks communities are a well-studied research topic. However, to date most research on this topic has focused on the problem of automatically detecting communities existing in a given social network. One of the most commonly used models for performing this task is the stochastic block model which can detect flat, hierarchical and overlapping communities (Peixoto 2019). In this work we assume the communities in question are known and do not require detection. We instead focus on the problem of modelling relationships existing between these communities. As such, the work presented in this paper is distinct from most existing research on this topic.

Modelling relationships existing between geographical regions is a well-studied problem in the field of geographical information science (Corcoran et al. 2012). The most cited model for performing this task is the intersection model proposed by Clementini et al. (1993) which models topological properties of relationships. As discussed later, this model provides inspiration for the model proposed in this work. Modelling the topological properties of graphs or networks is a well-studied problem in the fields of complex systems and network science. Models for performing this task commonly consider statistical features such as the vertex degree distribution (Corcoran and Mooney 2013) or algebraic features relating to connected components and holes or cycles (Corcoran 2020). The authors are unaware of any previous works that have considered the problem of modelling topological relationships existing between social network communities.

Valente (2012) defined four models of social network interventions. An *individual* intervention is a type of social network intervention where a set of individuals are identified and their characteristics are altered. This type of intervention is commonly implemented by selecting a set of individuals to act as influencers of other individuals in the social network. For example, Starkey et al. (2009) used this approach when implementing an intervention to reduce the practice of smoking. A *segment* or *community* intervention is a type of social network intervention where a set of individuals sharing a given characteristic are identified and their behaviours are altered. For example, Buller et al. (1999) used this type of intervention to increase fruit and vegetable intake among lower socioeconomic, multicultural labour and trades employees. An *induction* or *relationship* intervention is a type of social network intervention where a set of relationships existing between individuals are identified and the features of these relationships are altered. For example, Hoffman et al. (2013) used this type of intervention to perform peer education of HIV prevention. Finally, an *alteration* intervention is a type of social network intervention where individuals and relationships are added and deleted from the social network (Wilder et al. 2018). For example, Litt et al. (2007) used this type of intervention to help treat alcoholics by reducing their network support for drinking.

A social network intervention which is defined with respect to an event, such as a terrorist attack or an election, is known as an *event-based intervention*. Such interventions can take place before, during or after the event in question. Innes et al. (2021) examined how event-based interventions can influence public understandings and definitions of terrorist attacks after they happen. The authors identified three corresponding models of social network interventions associated with the communication of misinformation and disinformation. They entitled these models *spoofing*, *truthing* and *social proofing*. Spoofing involves the use of trickery, deception or misdirection to misrepresent the identity of sources and/or the validity and reliability of information. Truthing involves the use of truth claims, including conspiratorial hidden truths, or presenting factual information to try and persuade. Finally, social proofing involves the use of feedback mechanisms to construct an aura (sometimes illusion) of social support to influence the behaviour of others. Dobreva et al. (2020) examined how event-based interventions which considered the dissemination of ‘soft facts’ were used to influence the public before and after the UK Brexit referendum. The authors define soft facts as facts where the corresponding information provenance is uncertain and include rumours, conspiracy theories and propaganda.

Based on the above review of related works, we believe that the proposed model of social network interventions represents a novel combination of research ideas from the domains of social network analysis, topology and geographical information science.

## Social network intervention model

In this section we present the proposed model of social network interventions which considers topological relationships between communities. The proposed model provides a framework for both defining and determining an implementation of a social network intervention. In this context, defining an intervention means to state the desired state of the social network one wishes to achieve. On the other hand, determining the implementation of an intervention means to determine a sequence of steps which achieves this state. In the following three subsections we present in turn a model of social networks, a model of topological relationships and a model for both defining and determining the implementation of a social network intervention.

### Social network

Let ${\mathcal {G}}$ denote the space of graphs, *L* denote a set of community types and ${\mathcal {C}}$ denote the space of mappings from *L* to ${\mathcal {G}}$. We model a social network as a graph $G=(V,E) \in {\mathcal {G}}$ plus a mapping $C \in {\mathcal {C}}$. *V* is a set of elements called *vertices* which model individuals and *E* is a set of pairs of vertices called *edges* which model relationships existing between pairs of individuals. Note that, the type of relationship modelled by a given social network is a function of the application in question, but commonly modelled relationships are friendship, social influence and information/resource flow. A vertex may belong to zero, one or more than one community. The map *C* is a mapping from community type to the corresponding vertex-induced subgraph of the community in question. Note that, we assume crisp as opposed to fuzzy community membership. This choice is motivated by the fact that most social network data have this property.

To illustrate this model consider again the example social network illustrated in Fig. 1(a) which contains two communities represented by blue and red regions. Let the community types in question be ‘blue’ and ‘red,’ respectively. Given this, the social network in question is modelled as:

1 $$\begin{aligned} \begin{aligned} V&= \lbrace a,b,c,d,e \rbrace \\ E&=\lbrace (a,b), (a,c), (b,c), (c,d), (d,e) \rbrace \\ L&=\lbrace \text {`blue'}, \text {`red'} \rbrace \\ C(\text {`blue'})&= (\lbrace a,b,c \rbrace , \lbrace (a,b), (a,c), (b,c) \rbrace ) \\ C(\text {`red'})&= (\lbrace c,d,e \rbrace , \lbrace (c,d), (d,e) \rbrace ) \end{aligned} \end{aligned}$$

**Fig. 2:**
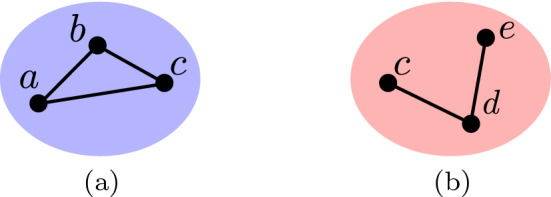
The mappings $C(\text {`blue'})$ and $C(\text {`red'})$ corresponding to the social network of Fig. 1(**a**) and Eq. 1 are displayed in (**a**) and (**b**), respectively (colour figure online)

The mappings $C(\text {`blue'})$ and $C(\text {`red'})$ corresponding to this model are illustrated in Fig. 2(a) and (b) respectively.

The above model of social networks is general in nature and can be used to model many different types of real-world social networks. Later we demonstrate this by considering three real-world social networks and associated problems in the domains of health and security.

### Topological relationships

**Fig. 3:**
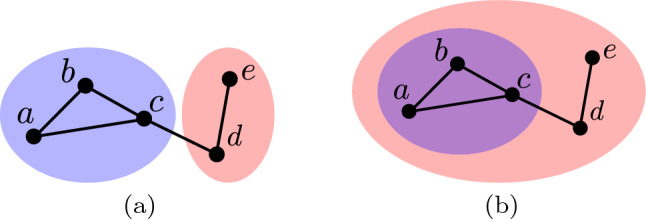
Hypothetical social networks containing blue and red communities are displayed in (**a**) and (**b**). The communities exhibit topological relationships of *disjoint* and *containment*, respectively (colour figure online)

Modelling topological relationships is a challenging problem. Given a social network, simply modelling topological properties of the corresponding graph cannot distinguish between different topological relationships. For example, consider the topological relationships of disjoint and containment illustrated in Fig. 3(a) and (b), respectively. Despite having different topological relationships, the graphs in question are identical and therefore have identical topological properties.

To overcome this challenge we define a set of subgraphs which are a function of the relationship in question. Specifically, these subgraphs are defined using a set of mappings *I*, *U* and *S* which have the form ${\mathcal {G}} \times {\mathcal {G}} \rightarrow {\mathcal {G}}$. We refer to these mappings as *binary relations*. For example, the binary relation *I* corresponds to the intersection of the communities in question. The use of binary relations is motivated by the intersection model proposed by Clementini et al. (1993) for modelling topological relationships between geographical regions. In this model relationships are modelled by defining a set of regions which are a function of the relationship in question.

Given the above set of binary relations, we model the topological relationship in question by modelling topological properties of these relations. Specifically, we define a mapping $T: {\mathcal {G}} \rightarrow {\mathcal {Z}}$ which is applied to each binary relation where ${\mathcal {Z}}$ denotes the set of multisets of positive integers. In this context, an element of ${\mathcal {Z}}$ models the number and size of connected components contained in the binary relation in question. The use of multisets to model topological properties is motivated by persistent homology which is a model of topological properties from the field of applied topology (Edelsbrunner and Harer 2010). In the remainder of this section we describe in detail the above model of topological relationships and demonstrate that it can make useful distinctions between topological relationships.

The specific binary relations one considers are determined by the application in question and in turn what topological properties one is attempting to model. In this work we consider the three binary relations *I*, *U* and *S* which we found to be sufficient for making useful distinctions between topological relationships. The binary relation *I* is defined in Eq. 2 where $g \, \cap \, g'$ denotes the graph containing the intersection of the vertex and edge sets corresponding to *g* and $g'$. For example, the binary relation $I(C(\text {`blue'}), C(\text {`red'}))$ between the communities illustrated in Fig. 1(a) and (b) is the graphs $(\lbrace a \rbrace , \lbrace \rbrace )$ and $(\lbrace \rbrace , \lbrace \rbrace )$, respectively.

2 $$\begin{aligned} \begin{aligned} I: {\mathcal {G}} \times {\mathcal {G}}&\rightarrow {\mathcal {G}} \\ g \times g'&\mapsto g \, \cap \, g' \end{aligned} \end{aligned}$$

The binary relation *U* is defined in Eq. 3 where $g \, \cup \, g'$ denotes the graph containing the union of the vertex and edge sets corresponding to *g* and $g'$. For example, the binary relation $U(C(\text {`blue'}), C(\text {`red'}))$ between the communities illustrated in Fig. 1(a) and (b) is the graphs $(\lbrace a,b,c,d,e \rbrace , \lbrace (a,b), (a,c), (b,c), (c,d), (d,e) \rbrace )$ and $(\lbrace a,b,d,e \rbrace , \lbrace (a,b), (d,e) \rbrace )$, respectively.

3 $$\begin{aligned} \begin{aligned} U: {\mathcal {G}} \times {\mathcal {G}}&\rightarrow {\mathcal {G}} \\ g \times g'&\mapsto g \, \cup \, g' \end{aligned} \end{aligned}$$

The binary relation *S* is defined in Eq. 3 where $g \, - \, g'$ denotes the graph containing vertex and edge sets corresponding to *g* less the vertex and edge sets, respectively, corresponding to $g'$. Note that, subtracting a vertex causes all adjacent edges to also be subtracted. For example, the binary relation $S(C(\text {`blue'}), C(\text {`red'}))$ between the communities illustrated in Fig. 1(a) and (b) is the graphs $(\lbrace a,b \rbrace , \lbrace (a,b) \rbrace )$ and $(\lbrace \rbrace , \lbrace \rbrace )$, respectively.

4 $$\begin{aligned} \begin{aligned} S: {\mathcal {G}} \times {\mathcal {G}}&\rightarrow {\mathcal {G}} \\ g \times g'&\mapsto g \, - \, g' \end{aligned} \end{aligned}$$

Given the three binary relations *I*, *U* and *S*, we model the topological relationship in question by modelling the topological properties of these relations. Specifically, the topological properties we model are the number of vertices in each connected component. This is achieved using the map *T* defined in Eq. 5 where ${\mathcal {Z}}$ denotes the set of multisets of positive integers and $g^c$ denotes the set of connected components contained in the graph *g*.

5 $$\begin{aligned} \begin{aligned} T: {\mathcal {G}}&\rightarrow {\mathcal {Z}} \\ g&\mapsto \lbrace \vert V' \vert : (V', E') \in g^c \rbrace \end{aligned} \end{aligned}$$

**Fig. 4:**
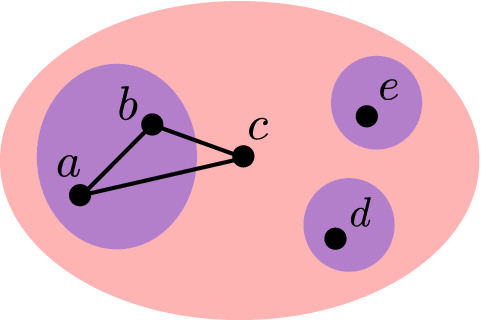
A hypothetical social network containing blue and red communities is displayed. The communities exhibit a topological relationship of *containment* (colour figure online)

To illustrate the map *T* consider the social network displayed in Fig. 4 containing blue and red communities which exhibit the topological relationship of containment. The binary relation *I* of these communities is a graph containing three connected components of sizes two, one and one. Therefore, the map *T* of this binary relation is the multiset $\lbrace 2,1,1 \rbrace$. This inference is formally stated as follows:

6 $$\begin{aligned} \begin{aligned} I(C(\text {`blue'}), C(\text {`red'}))&= (\lbrace a,b,e,d \rbrace , \lbrace (a,b) \rbrace ) \\ T((\lbrace a,b,e,d \rbrace , \lbrace (a,b) \rbrace ))&= \lbrace 2, 1, 1 \rbrace \end{aligned} \end{aligned}$$

Composing the set of binary relations with the map *T* we define the set of maps $T \circ I$, $T \circ U$ and $T \circ S$ as follows:

7 $$\begin{aligned} \begin{aligned} T \circ I: {\mathcal {G}} \times {\mathcal {G}}&\rightarrow {\mathcal {Z}} \\ g \times g'&\mapsto T(g \, \cap \, g') \end{aligned} \end{aligned}$$

8 $$\begin{aligned} \begin{aligned} T \circ U: {\mathcal {G}} \times {\mathcal {G}}&\rightarrow {\mathcal {Z}} \\ g \times g'&\mapsto T(g \, \cup \, g') \end{aligned} \end{aligned}$$

9 $$\begin{aligned} \begin{aligned} T \circ S: {\mathcal {G}} \times {\mathcal {G}}&\rightarrow {\mathcal {Z}} \\ g \times g'&\mapsto T(g \, - \, g') \end{aligned} \end{aligned}$$

The maps $T \circ I$ and $T \circ U$ are symmetric, while the map $T \circ S$ is not. That is, $T \circ I(a, b) = T \circ I(b, a)$ while $T \circ S(a, b) \ne T \circ S(b, a)$. Therefore, given two communities *a* and *b* we model the topological relationship in question using the set of maps $T \circ I(a, b)$, $T \circ U(a, b)$, $T \circ S(a, b)$ and $T \circ S(b, a)$. To illustrate this model consider again the social network displayed in Fig. 4. In this example the topological relationship existing between the blue and red communities is modelled by:

10 $$\begin{aligned} \begin{aligned} T \circ I(C(\text {`blue'}), C(\text {`red'}))&= \lbrace 2,1,1 \rbrace \\ T \circ U(C(\text {`blue'}), C(\text {`red'}))&= \lbrace 3,1,1 \rbrace \\ T \circ S(C(\text {`blue'}), C(\text {`red'}))&= \lbrace \rbrace \\ T \circ S(C(\text {`red'}), C(\text {`blue'}))&= \lbrace 1 \rbrace \end{aligned} \end{aligned}$$

**Fig. 5:**
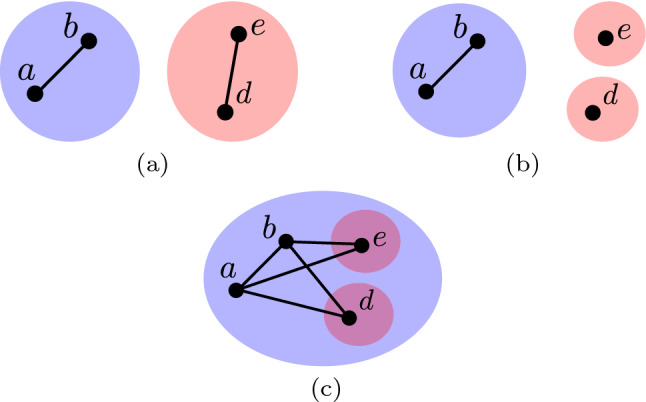
The topological relationship between the communities changes from *disjoint* in (**a**) and (**b**) to *containment* in (**c**)

The map *T* models both the number and size of connected components. Modelling the size of connected components is necessary for solving many problems involving social network interventions. To illustrate this point consider a social network which contains one community corresponding to a terrorist group and a second community corresponding to undercover agents working for a government intelligence agency. Furthermore, let us say these communities are modelled by the social network illustrated in Fig. 5(a) where individuals belonging to the former community lie in the blue coloured region, while individuals belonging to the latter community lie in the red coloured region. The objective of the undercover agents is to secretly infiltrate the terrorist community in a manner which minimises the probability that the discovery of one agent by the terrorist community causes the discovery of the other. Achieving this goal may be posed as transforming the topological relationship in question as follows. First the connection between the agents is removed, so the agents become disjoint as illustrated in Fig. 5(b). That is, the size of each connected component in the agents community is one. Next, the agents become members of, or contained within, the terrorist community as illustrated in Fig. 5(c). This example illustrates that interventions with multiple objectives can be modelled using the proposed approach by either performing a series of individual interventions or defining a single desired topological relationship which models all objectives.

Finally, one does not need to consider the full set of binary relations defined above to model many topological relationships. For example, consider the topological relationship corresponding to disjoint. This topological relationship can be modelled using only the binary relation *I* which corresponds to the intersection of the communities in question. Specifically, the topological relationship is disjoint if and only if $T \circ I$ equals the empty set. That is, this is a necessary and sufficient condition.

### Defining and implementing interventions

In this section we describe how the proposed model of topological relationships can be used to define and determine the implementation of a social network intervention. Recall that, defining an intervention means to state the desired topological relationship one wishes the social network communities in question to have. On the other hand, determining the implementation of an intervention means to determine a sequence of operations which if applied achieves this topological relationship. For example, consider again the social network displayed in Fig. 1(a) where the desired topological relationship is disjoint communities. A sequence of operations which achieves this relationship is the removal of vertex *a*, and the result of applying this operation is displayed in Fig. 1(b).

The remainder of this section is structured as follows. In Sect. 3.3.1 we define a set of operations which can be applied to a social network. In Sect. 3.3.2 we describe how an intervention can be formally defined in terms of a corresponding set of necessary and sufficient conditions. If these conditions are satisfied, this implies the intervention in question has been successfully implemented. Finally, in Sect. 3.3.3 we describe how the implementation of the intervention can be determined where this implementation is defined in terms of the above operations. The above conditions and implementation for a given intervention are both determined automatically. Hence, this allows the process of reasoning about interventions to be performed completely at the abstraction of topological relationships.

#### Social network operations

Recall that ${\mathcal {G}}$ denotes the space of graphs, *L* denotes a set of community types and ${\mathcal {C}}$ denotes the space of mappings from *L* to ${\mathcal {G}}$. Furthermore, recall that we model a social network as an element of ${\mathcal {G}}$ plus an element of ${\mathcal {C}}$. Let $2^L$ denote the power set of *L* (the set of all subsets of *L*). Given a social network $G=(V,E) \in {\mathcal {G}}$ and $C: L \rightarrow {\mathcal {G}} \in {\mathcal {C}}$, we define the following set of operations:

11 $$\begin{aligned} \begin{aligned} V^-:V&\rightarrow {\mathcal {G}} \\ E^+:V&\times V \rightarrow {\mathcal {G}} \\ E^-:E&\rightarrow {\mathcal {G}} \\ C^\Delta :V&\times 2^L \rightarrow {\mathcal {C}} \end{aligned} \end{aligned}$$

**Fig. 6:**
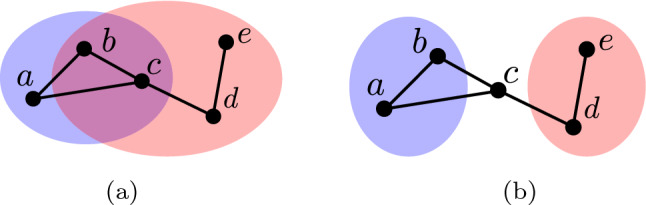
Applying the operations $C^\Delta (b, \lbrace \text {`blue'}, \text {`red'} \rbrace )$ and $C^\Delta (c, \lbrace \rbrace )$ to the social network displayed in Fig. 1(a) results in the social networks displayed in (a) and (b), respectively (colour figure online)

The operation $V^-$ removes a specified vertex from the graph *G*. Note that, removing a vertex results in all edges adjacent to the vertex also being removed. For example, applying the operation $V^-(c)$ to the social network displayed in Fig. 1(a) results in the social network displayed in Fig. 1(b). The operation $E^+$ adds an edge between a specified pair of vertices to the graph *G*. The operation $E^-$ removes a specified edge from the graph *G*. The operation $C^\Delta$ assigns a given vertex to a given set of communities and in turn changes the mapping *C*. For example, applying the operations $C^\Delta (b, \lbrace \text {`blue'}, \text {`red'} \rbrace )$ and $C^\Delta (c, \lbrace \rbrace )$ to the social network displayed in Fig. 1(a) results in the social networks displayed in Figs 6(a) and (b), respectively.

How each of the operations defined above is performed in reality and not in the abstract graph model will depend on the real-world application domain in question. For example, consider the operation of removing a vertex which corresponds to removing an individual from the social network. In some application domains this may be performed by detaining the individual, while in other application domains it may be performed by confiscating the individual’s mobile communication device. Furthermore, the usefulness of each operation will also depend on the application domain in question. For example, removing a vertex which corresponds to removing an individual from the social network, possibly through detainment, may be very difficult or impossible to perform in a particular application domain. To model these characteristics of the problem, we define a set of *feasible operations* which are a subset of the operations defined above. These operations correspond to those which can be successfully performed and are specific to the application domain in question.

Note that, the set of operations defined above are very similar to the set of intervention models originally proposed by Valente (2012). For example, an individual intervention which alters the characteristics of individuals is similar to the operation $C^\Delta$. Likewise, an alteration intervention which alters the structure of the social network is similar to the operations $V^-$, $E^-$ and $E^+$.

#### Defining interventions

A social network intervention can be formally defined in terms of a corresponding set of necessary and sufficient conditions. For example, consider again the social network in Fig. 1(a) where we wish to perform an intervention which transforms the topological relationship to disjoint communities. A necessary and sufficient condition for the topological relationship of disjoint is defined as follows which states that the intersection of the communities in question is empty:

12 $$\begin{aligned} T \circ I(C(\text {`blue'}), C(\text {`red'})) = \lbrace \rbrace \end{aligned}$$

**Table 1:** A set of topological relationships plus corresponding necessary and sufficient conditions are provided in this table. *A* and *B* correspond to vertex-induced subgraphs of two communities

Topological relationship	Necessary and sufficient conditions
between communities *A* and *B*	
A and B disjoint	$T \circ I(A, B) = \lbrace \rbrace$
A contained in B	$T \circ S(A, B) = \lbrace \rbrace$
B contained in A	$T \circ S(B, A) = \lbrace \rbrace$
A and B equal	$T \circ S(A, B) = \lbrace \rbrace$
	$$T \circ S(B, A) = \lbrace \rbrace$$
A and B partial overlap	$T \circ I(A, B) \ne \lbrace \rbrace$
	$$T \circ S(A, B) \ne \lbrace \rbrace$$
	$$T \circ S(B, A) \ne \lbrace \rbrace$$
A and B touch	$T \circ I(A, B) = \lbrace \rbrace$
	$$\vert T \circ U(A, B) \vert < \vert T(A) \vert + \vert T(B) \vert$$

There exist many different topological relationships which may exist between social network communities. Table 1 lists some possible topological relationships plus corresponding necessary and sufficient conditions. The last row in this table provides the necessary and sufficient conditions corresponding to the topological relationship of touching. We describe a topological relationship as touching if no vertex is a member of both communities in question, but there exists one or more edges connecting the vertices of these communities. For example, the communities displayed in Fig. 3(a) have this topological relationship because the vertices *c* and *d*, which belong to the blue and red communities, respectively, are connected.

For most topological relationships in the above table, it is self-evident why the corresponding conditions are necessary and sufficient. Therefore, here we only present a proof of the necessary and sufficient conditions for the topological relationship of touching. This proof is provided in Theorem 1 where $\vert . \vert$ denotes the multi-set cardinality function.

##### Theorem 1

Necessary and sufficient conditions for the topological relationship of touch between communities *A* and *B* are $T \circ I(A, B) = \lbrace \rbrace$ and $\vert T \circ U(A, B) \vert < \vert T(A) \vert + \vert T(B) \vert$.

##### Proof

To prove the conditions are necessary and sufficient we prove that the topological relationship exists if and only if the conditions are satisfied.

We first prove that, if the topological relationship exists, the conditions are satisfied. If the topological relationship is touch, then by definition communities are disjoint and the condition $T \circ I(A, B) = \lbrace \rbrace$ is satisfied. Furthermore, if the communities are connected by edges, the number of connected components in the union will be less than the sum of the number of connected components in each community.

Next we prove that if the conditions are satisfied, the topological relationship exists. If the conditions are satisfied, then the communities are disjoint. Furthermore, if the number of connected components in the union is less than the sum of the number of connected components in each community, then the communities are connected by edges. $\square$

#### Implementing interventions

In this section we consider the process of determining the implementation of a social network intervention. Recall that an implementation corresponds to a sequence of feasible operations which if applied achieves the necessary and sufficient conditions of the intervention in question. The algorithm used to determine an implementation will vary depending on the intervention in question and the set of feasible operations. In the remainder of this section we demonstrate the process of determining the implementation of a specific intervention.

To demonstrate the process for determining the implementation of an intervention consider again the social network in Fig. 1(a) where we wish to perform an intervention which transforms the topological relationship to disjoint communities. By examining Table 1, we see that the necessary and sufficient conditions corresponding to the topological relationship of disjoint are $T \circ I(\text {`blue'}, \text {`red'}) = \lbrace \rbrace$. If we assume all operations in Eq. 11 are feasible, we can determine multiple different implementations for this intervention. One implementation can be determined using Algorithm 1. This algorithm first determines the graph corresponding to the intersection of both communities (line 2). It next applies the operation $V^-$ to all vertices in this graph (lines 2 to 5).
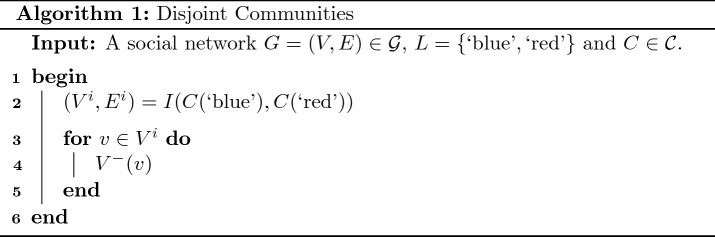


Applying Algorithm 1 to the social network in Fig. 1(a), returns an implementation which contains the single operation $V^-(c)$ which removes the vertex *c* from the social network. The result of applying this implementation is displayed in Fig. 1(b). In Theorem 2 we prove that this method for determining an implementation of the intervention in question generalises to all social networks.

##### Theorem 2

Consider a social network where $G=(V,E) \in {\mathcal {G}}$, $L =\lbrace \text {`blue'}, \text {`red'} \rbrace$ and $C \in {\mathcal {C}}$ plus a set of feasible operations containing $V^-$. Algorithm 1 determines an implementation of the intervention which transforms the topological relationship between the communities in question to disjoint.

##### Proof

The necessary and sufficient conditions for the intervention in question are $T \circ I(C(\text {`blue'}), C(\text {`red'})) = \lbrace \rbrace$. Algorithm 1 returns an implementation of the intervention which applies the operation $V^-$ to all vertices in the graph $I(C(\text {`blue'}), C(\text {`red'}))$. Applying this implementation will result in $I(C(\text {`blue'}), C(\text {`red'}))$ being an empty graph and in turn $T \circ I(C(\text {`blue'}), C(\text {`red'})) = \lbrace \rbrace$. Hence the necessary and sufficient conditions are satisfied. $\square$

An alternative implementation of the above intervention can be determined by applying the operation $C^\Delta (., \lbrace \text {`blue'} \rbrace )$ to all vertices in the graph $T \circ I(C(\text {`blue'}), C(\text {`red'}))$. In the context of this example, this results in an implementation containing the single operation $C^\Delta (c, \lbrace \text {`blue'} \rbrace )$ which removes the vertex *c* from the red community.

Given multiple implementations of an intervention, in many cases one may wish to select a single implementation to apply. The actual implementation selected will in many cases depend on the real-world application domain in question. One implementation may be less difficult or expensive to implement in the application domain. In that case it makes sense to select that implementation. For example, removing an edge which corresponds to removing a communication link in the application domain may be less difficult to implement than removing a vertex which corresponds to removing an individual in the application domain. Furthermore, one implementation may have a higher probability of being successful in a given application domain. Again, in that case it makes sense to select that implementation. Badham et al. (2021) performed an analysis of social network interventions involving the diffusion of behaviour change. The authors found that the success of such interventions is strongly affected by the connectivity structure of the network.

Note that, the methods described above for defining and implementing interventions assume that while they are being performed, the social network in question does not undergo any changes other than those specified by the implementation. If this were to occur, the definition and implementation in question may need to be redetermined. Furthermore, if the social network underwent changes after the intervention was successfully performed, again the definition and implementation in question may need to be redetermined.

## Simulation of social network interventions

In this section we demonstrate that the proposed model of social network interventions, which models topological relationships between communities, provides a useful abstraction for solving many real-world problems. In the following subsections we consider three specific social networks and associated problems in the domains of health and security. Two of these correspond to hypothetical social networks, while the third corresponds to a real-world social network. The interventions in question are performed in simulation which is a standard practice in the field of social network research (Badham et al. 2018).

Using the above problems, we demonstrate that the proposed model makes the process of reasoning about intervention less complex than previous models which directly model vertices, edges and individual communities. For a given problem, the intervention in question is first defined in terms of a desired topological relationship. As described in Sect. 3, this definition maps to a set of necessary and sufficient conditions which in turn maps to an implementation corresponding to a sequence of feasible operations. Since both of these mappings are determined automatically, this allows the process of reasoning about interventions to be performed completely at the abstraction of topological relationships.

### Infiltrate a terrorist group

In this section we describe a social network intervention in the security domain with respect to a hypothetical social network. Consider again the social network displayed in Fig. 5(a) where the blue community corresponds to a terrorist group and the red community corresponds to undercover agents working for a government. Recall that, the undercover agents wish to secretly infiltrate the terrorist community in a manner which minimises the probability that the discovery of one agent by the terrorist community causes the discovery of the other.

In the context of this domain we assume the following three feasible operations. Firstly, we assume the operation $E^-$, which removes a given edge, can be applied to an edge if and only if the adjacent vertices correspond to agents. This is a reasonable assumption given the intervention in question is being carried out by the agents. Secondly, we assume the operation $C^\Delta$, which changes the community membership of a given vertex, can be applied to a vertex if and only if the vertex corresponds to an agent. This is a reasonable assumption given the agents are trained in the art of infiltrating terrorist groups. For example, performing the operation $C^\Delta (d, \lbrace \text {`blue'}, \text {`red'} \rbrace )$ would involve the agent *d* successfully hiding their membership of the agent community from members of the terrorist community. Finally, we assume the operation $E^+$, which adds an edge between pairs of vertices, can be applied if and only if one of the vertices in question corresponds to an agent. Again, this is a reasonable assumption given the agents are trained in the art of infiltrating terrorist groups.

Successfully infiltrating the terrorist community can be achieved by applying two interventions which we now describe. In the first intervention the desired topological relationship between the communities is disjoint agents who are not members of the terrorist group. Let $\lbrace 1, \dots ,1 \rbrace$ denote a multiset containing only elements equal to 1. Necessary and sufficient conditions for this relationship are:

13 $$\begin{aligned} \begin{aligned} T (C(\text {`red'}))&= \lbrace 1, \dots ,1 \rbrace \\ T \circ I(C(\text {`blue'}), C(\text {`red'}))&= \lbrace \rbrace \end{aligned} \end{aligned}$$

The first condition states that each agent is not connected to any other agent, while the second condition states that the two communities are disjoint.

If we assume the two communities initially have the topological relationship disjoint, an implementation of this intervention can be determined using Algorithm 2. The first step in this implementation determines the red community corresponding to agents (line 2). Subsequently, each edge between vertices in this community is removed using the operation $E^-$ (lines 3 to 5).
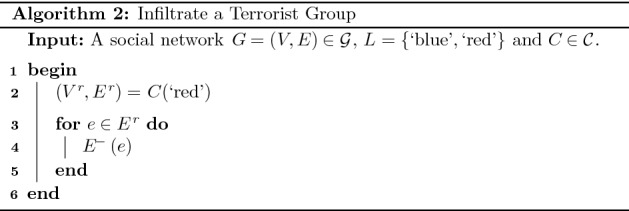


Applying the above algorithm to the social network displayed in Fig. 5(a) results in an implementation containing the single operation $E^-((e,d))$ which removes the edge (*e*, *d*) from the social network. Figure 5(b) displays the result of applying this implementation. In Theorem 3 we prove that this method for determining an implementation of the intervention in question generalises to all social networks.

#### Theorem 3

Consider a social network where $G=(V,E) \in {\mathcal {G}}$, $L =\lbrace \text {`blue'}, \text {`red'} \rbrace$ and $C \in {\mathcal {C}}$ plus a set of feasible operations containing $E^-$. If we assume that initially the communities have the topological relationship disjoint, Algorithm 2 determines an intervention implementation which satisfies the necessary and sufficient conditions in Eq. 13.

#### Proof

Initially the communities have the topological relationship disjoint; therefore, the first condition in Eq. 13 is immediately satisfied. Applying the operation $E^-$ to all edges in the graph $C(\text {`red'})$ results in this graph having a set of connected components each of size one. Hence, the second condition in Eq. 13 is satisfied. $\square$

In the second intervention the desired topological relationship between the communities is disjoint agents who are members of the terrorist group. Necessary and sufficient conditions for this relationship are the following:

14 $$\begin{aligned} \begin{aligned} T (C(\text {`red'}))&= \lbrace 1, \dots ,1 \rbrace \\ T \circ S(C(\text {`red'}), C(\text {`blue'}))&= \lbrace \rbrace \\ \vert T \circ U(C(\text {`red'}), C(\text {`blue'})) \vert&= 1 \end{aligned} \end{aligned}$$

The first condition states that each agent is not connected to any other agent. The second condition states that the agent community is contained in the terrorist community. The final condition states that $T \circ U(C(\text {`red'}), C(\text {`blue'}))$ is a single connected component and in turn that each agent is connected either directly or indirectly to each member of the terrorist community.

An implementation of this intervention can be determined using Algorithm 3. The first step in this implementation is to determine the red and blue communities corresponding to agents and terrorists, respectively (lines 2 and 3). Next, the community membership of each vertex in the red community is changed to $\lbrace \text {`blue'}, \text {`red'} \rbrace$ using the operation $C^\Delta$ (lines 4 to 6). Finally, an edge is added between each pair of vertices in the red and blue communities (lines 7 to 11).
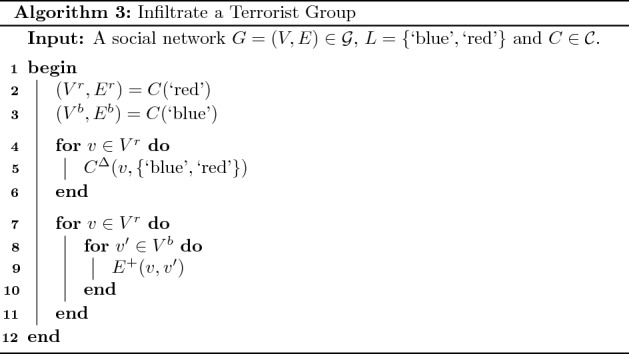


Applying this algorithm to the social network displayed in Fig. 5(b) results in an implementation containing the sequence of operations $C^\Delta (e, \lbrace \text {`blue'}, \text {`red'} \rbrace )$, $C^\Delta (d, \lbrace \text {`blue'}, \text {`red'} \rbrace )$, $E^+(d,a)$, $E^+(d,b)$, $E^+(e,a)$ and $E^+(e,b)$. Figure 5(c) displays the result of applying this implementation. In Theorem 4 we prove that this method for determining an implementation of the intervention in question generalises to all social networks.

#### Theorem 4

Consider a social network where $G=(V,E) \in {\mathcal {G}}$, $L =\lbrace \text {`blue'}, \text {`red'} \rbrace$ and $C \in {\mathcal {C}}$ plus a set of feasible operations containing $C^\Delta$ and $E^+$. If we assume that initially the conditions in Eq. 13 are satisfied, Algorithm 3 determines an intervention implementation which satisfies the necessary and sufficient conditions in Eq. 14.

#### Proof

Initially the conditions in Eq. 13 are satisfied; therefore. the first condition in Eq. 14 is immediately satisfied. Applying the operation $C^\Delta (., \lbrace \text {`blue'}, \text {`red'} \rbrace )$ to all vertices in the red community will result in $S(C(\text {`red'}), C(\text {`blue'}))$ being an empty graph and in turn $T \circ S(C(\text {`red'}), C(\text {`blue'})) = \lbrace \rbrace$. Applying the operation $E^+$ to each pair of vertices in the red and blue communities will result in $U(C(\text {`red'}), C(\text {`blue'}))$ being a single connected component and in turn $\vert T \circ U(C(\text {`red'}), C(\text {`blue'})) \vert = 1$. $\square$

### Reducing the spread of HIV

**Fig. 7:**
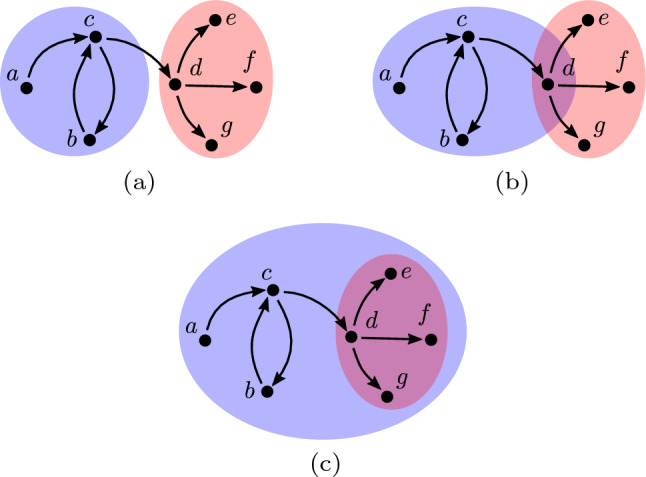
The topological relationship between the communities changes from *disjoint* in (**a**) to *partial overlap* in (**b**) and to *containment* in (**c**)

In this section we describe a social network intervention in the health domain with respect to a hypothetical social network. A common social network intervention concerns attempting to influence members of, or spread information within, a social network (Smit et al. 2021). This type of intervention can be modelled in terms of topological relationships. To illustrate this consider the two communities displayed in Fig. 7(a) where the blue community corresponds to a particular set of individuals who have a high risk of HIV infection (e.g. men who have sex with men and do not practice HIV prevention techniques), while the red community corresponds to a set of individuals educated in HIV preventing techniques (e.g. use of condoms, limiting number of sexual partners). In this graph a directed edge represents the presence of *assimilative social influence* whereby the source individual influences the target individual towards reducing differences (Flache et al. 2017).

The topological relationship between the communities in question is disjoint, and this may be considered a contributing factor to the existence of the high-risk community. Modelling the topological relationship between the communities allows the automated detection of such contributing factors. Furthermore, applying an intervention which alters the topological relationship so that the red community is contained in the blue community would eliminate this factor. A necessary and sufficient condition for this topological relationship is:

15 $$\begin{aligned} T \circ S(C(\text {`red'}), C(\text {`blue'})) = \lbrace \rbrace \end{aligned}$$

If we assume a set of feasible operations containing the operation $C^\Delta$, an implementation of this intervention can be determined using Algorithm 4. The first step in this implementation defines a new community, which we call the green community, containing highly influential members of the red community (line 2). Next, the community membership of each vertex in this community is changed to $\lbrace \text {`red'}, \text {`blue'} \rbrace$ using the operation $C^\Delta$ (lines 3 to 5). This operation in turn has the effect of, through influence, changing the community membership of other vertices in the red community to $\lbrace \text {`red'}, \text {`blue'} \rbrace$.
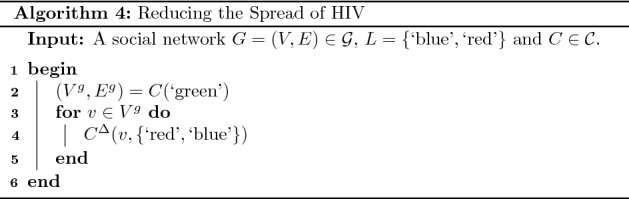


Applying this algorithm to the social network displayed in Fig. 7(a) results in an implementation containing the single operation $C^\Delta (d, \lbrace \text {`red'}, \text {`blue'} \rbrace )$. Figure 7(b) displays the result of applying this implementation. This operation in turn has the effect of changing the community membership of all other vertices in the red community to $\lbrace \text {`red'}, \text {`blue'} \rbrace$. This is displayed in Fig. 7(c).

In Theorem 5 we prove that this method for determining an implementation of the intervention in question generalises to all social networks.

#### Theorem 5

Consider a social network where $G=(V,E) \in {\mathcal {G}}$, $L =\lbrace \text {`blue'}, \text {`red'} \rbrace$ and $C \in {\mathcal {C}}$ plus a set of feasible operations containing $C^\Delta$. If we assume a highly influential green community which is a subset of the red community and directly connect to all other vertices in this community, Algorithm 4 determines an intervention implementation which satisfies the necessary and sufficient condition in Eq. 15.

#### Proof

Applying the operation $C^\Delta (., \lbrace \text {`red'}, \text {`blue'} \rbrace )$ to each vertex in the green community will change the community membership in question to $\lbrace \text {`red'}, \text {`blue'} \rbrace$. Since the green community is directly connected to all other vertices in the red community, this will have the effect of changing the community membership of all other vertices in this community to $\lbrace \text {`red'}, \text {`blue'} \rbrace$. $\square$

### Reducing network support for drinking

**Fig. 8:**
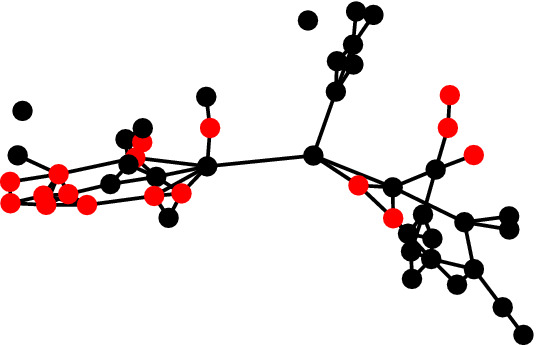
Students belonging to the frequent and infrequent drinker communities are represented by red and black vertices, respectively. Edges represent the existence of friendship relationships between the vertices in question (colour figure online)

In this section we describe a social network intervention in the health domain with respect to a real-world social network. Michell and Amos (1997) collected a social network where vertices correspond to students in a school in Scotland and edges correspond to the existence of a friendship. Each student was asked to indicate if they consume alcohol more than once a week, once a week, once a month, once or twice a year or never. This social network was collected at three different time points. For the purposes of this work we consider the social network collected at the second time point and a excerpt containing fifty students which can be downloaded from the Internet^1^. We consider two communities in this social network corresponding to frequent and infrequent drinkers. Specifically, the first community corresponds to those students who drink once a week or more than once a week, while the second community corresponds to all remaining students. This social network is illustrated in Fig. 8 where students belonging to the frequent and infrequent drinker communities are represented by red and black vertices, respectively.

The network support for drinking refers to the degree to which an individual’s social connections encourage drinking (Litt et al. 2007). Network support for drinking has been found to be predictive of drinking behaviour (Havassy et al. 1991; Ivaniushina and Titkova 2021). In fact, studies have found that social network interventions which alter the network support for drinking can improve drinking behaviour (Litt et al. 2007). We now demonstrate how the model proposed in this work provides a useful abstraction for performing such interventions.

Let $G=(V,E) \in {\mathcal {G}}$ denote the social network graph in question. Also, let $L =\lbrace \text {`frequent'}, \text {`infrequent'} \rbrace$ denote the set of community types in question corresponding to frequent and infrequent drinkers. Let *N* be the following map from a vertex to its corresponding ego community:

16 $$\begin{aligned} \begin{aligned} N: V&\rightarrow {\mathcal {G}} \\ v&\mapsto ( \lbrace v \rbrace \cup \lbrace v': (v,v') \in E \rbrace , \lbrace \rbrace ) \end{aligned} \end{aligned}$$

**Fig. 9:**
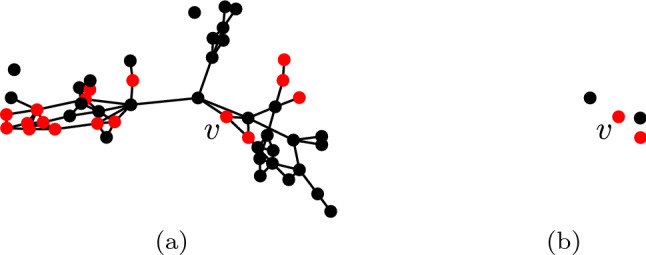
A social network is displayed in (**a**) where a single vertex *v* is indicated. The corresponding ego community *N*(*v*) is displayed in (**b**)

Informally, the ego community of a vertex *v* is the graph containing *v* plus all vertices adjacent to *v*. Consider the social network displayed in Fig. 9(a) where a single vertex *v* is indicated. The corresponding ego community *N*(*v*) contains four vertices and is displayed in Fig. 9(b).

Let us assume that the network support for drinking can be reduced by performing an intervention such that each vertex either belongs to the infrequent community or is directly connected to a vertex belonging to this community. A necessary and sufficient condition for this topological relationship is:

17 $$\begin{aligned} \vert T \circ I(N(v), C(\text {`infrequent'})) \vert \ge 1 \, , \, \forall \, v \in V \end{aligned}$$

For example, the value $\vert T \circ I(N(v), C(\text {`infrequent'})) \vert$ corresponding to the vertex *v* in Fig. 9(a) is 2. If we assume a set of feasible operations containing the operation $E^+$, an implementation of this intervention can be determined using Algorithm 5. The first step in this implementation determines the frequent and infrequent communities (lines 2 and 3). Next, for each vertex *v* in the frequent community where the above condition does not hold, a vertex $v'$ belonging to the infrequent community is selected uniformly at random and the operation $E^+(v,v')$ is applied (lines 4 to 9).
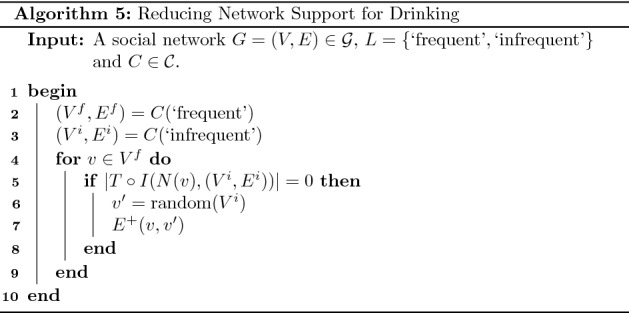

**Fig. 10:**
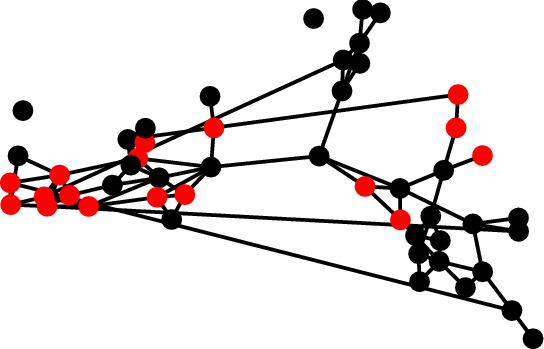
Students belonging to the frequent and infrequent drinker communities are represented by red and black vertices, respectively. Each vertex either belongs to the ‘infrequent’ community or is directly connected to a vertex belonging to this community (colour figure online)

This result of applying this intervention to the social network displayed in Fig. 8 is displayed in Fig. 10. The intervention in question added six additional edges and we can see that each vertex now either belongs to the infrequent community or is directly connected to a vertex belonging to this community. In Theorem 6 we prove that this method for determining an implementation of the intervention in question generalises to all social networks.

#### Theorem 6

Consider a social network where $G=(V,E) \in {\mathcal {G}}$, $L =\lbrace \text {`frequent'}, \text {`infrequent'} \rbrace$ and $C \in {\mathcal {C}}$ plus a set of feasible operation containing $E^+$. If we assume that initially the infrequent community contains one or more vertices, Algorithm 5 determines an intervention implementation which satisfies the necessary and sufficient conditions in Eq. 14.

#### Proof

For each vertex in the infrequent community, the condition in Eq. 14 is immediately satisfied. For each vertex in the frequent community for which this condition is not satisfied, an edge is added connecting the vertex in question to a random vertex in the infrequent community. This results in the condition in Eq. 14 being satisfied for each vertex in the infrequent community. $\square$

## Conclusions

In this work we proposed a novel model of social network interventions which considers topological relationships existing between communities. We subsequently demonstrated that this model provides an abstraction which in many cases is useful for solving problems involving social network interventions. To the authors’ knowledge, this is the first such model to consider topological relationships. As such, there exist many worthwhile directions for future research and development of the model. Some possible directions include the following.

The proposed model assumes a set of feasible operations which can be applied to a given social network to implement an intervention. This is an abstract view and does not consider how each operation is performed in reality. For example, removing a vertex from a social network may be performed in reality by detaining the individual in question or by confiscating their mobile communication device. The former approach may be feasible in the security domain if the individual is a known criminal and those performing the intervention are national police. However, in most domains detaining an individual is illegal. Therefore, it is important that the set of feasible operations is defined with respect to the application domain in question where how each operation is implemented in reality is clearly specified. Furthermore, the proposed model does not model the fact that different intervention implementations may have different probabilities of being successful. Modelling such probabilities would be useful in many situations. For example, where one has many possible implementations of a given intervention and wishes to select the one with the highest probability of success. The task of defining the intervention with highest probability of success could potentially be posed as an optimization problem.

The proposed model makes a distinction between defining and implementing an intervention where these tasks are performed independently. That is, one first defines an intervention and then determines how best to implement it. This may be a suboptimal approach whereby in many cases it may be beneficial to perform both tasks jointly. For example, by doing so one could define an intervention which both achieves a desired objective and is inexpensive to implement. The task of jointly defining and implementing an intervention which meets a specified objective could also potentially be posed as an optimization problem.

In this article we have considered a handful of specific applications in the domains of health and security. However the proposed model is very general in nature with many potential applications beyond these considered here. An interesting direction for future research would be to consider applications of the model to event-based interventions discussed in the related works section of this article.

Finally, in this article we have highlighted many potential benefits of using social network interventions. However, existing models generally only consider the direct consequences of interventions and fail to consider the potential for indirect or secondary consequences. These indirect consequences can potentially be very significant. For example, consider the interventions, of reducing face-to-face interactions, performed by many governments to reduce COVID-19 transmission. The indirect long-term impact of these interventions on the education of children due to school closures is not yet fully understood. Therefore, more research and improved modelling is needed in this space.

## Data Availability

The datasets generated during and/or analysed during the current study are available from the corresponding author on reasonable request.

## Footnotes

1 https://www.stats.ox.ac.uk/~snijders/siena/s50_data.htm.
